# Design and Performance Evaluation of Polymer Matrix Composite Helical Springs

**DOI:** 10.3390/polym14183900

**Published:** 2022-09-18

**Authors:** Ling Chen, Liwei Wu, Hongjun Fu, Youhong Tang

**Affiliations:** 1 ARC Training Centre for Green Chemistry in Manufacturing, Flinders University, Adelaide, SA 5042, Australia; 2 Institute for Nanoscale Science and Technology, College of Science and Engineering, Flinders University, Adelaide, SA 5042, Australia; 3 Innovation Platform of Intelligent and Energy-Saving Textiles, Tiangong University, Tianjin 300387, China; 4 School of Textiles Science and Engineering, Tiangong University, Tianjin 300387, China

**Keywords:** polymer matrix composite helical spring, polymer matrix composite spring wire rod, torsion, compression, resilience

## Abstract

Helical springs are indispensable mechanical parts widely used in industry. Lightweight is one of the development trends of helical springs. In this study, three kinds of lightweight polymer matrix composite helical springs (PMCHSs) with unidirectional, multistrand, and wrapped textile structural reinforcement (PMCHS-U, PMCHS-M, and PMCHS-W) were designed, manufactured, and evaluated. The performance of these PMCHSs and the relationship between their performance and their corresponding polymer matrix composite spring wire rods (PMCRs) were studied through the torsion test of the PMCRs and the compression and resilience tests of the PMCHSs. The results showed that the performance of the PMCHSs could be effectively improved by using the wrapped structure as the reinforcement. The compression capacity of PMCHS-W was 72.6% and 137.5% higher than that of PMCHS-M and PMCHS-U, respectively. The resilience performance of the PMCHSs decreased with the increase in the spring constant. The performances of the PMCHSs and a steel spring were compared. The results showed that the spring constant of the steel spring could be achieved when the masses of PMCHS-U, PMCHS-M, and PMCHS-W were only 75%, 63%, and 49% of the mass of the steel spring, respectively. This research is of great significance to the improvement in lightweight spring performance.

## 1. Introduction

A helical spring is an important part of shock absorption and cushioning in various industries [1,2]. The metal spring is heavy and easy to corrode, which limits its application. In recent years, the wide applications of polymer matrix composite materials with low density [3], high specific strength, high specific modulus [4,5], and corrosion resistance [6,7] have promoted the development of springs. In terms of practical applications, polymer matrix composite helical springs (PMCHSs) can be used as the suspension systems of tricycles, mountain bikes, motorcycles, automobiles, and trains, as well as in the aerospace field as the engine systems of aircraft and deployable antenna to receive and transmit signals [8]. PMCHSs can not only improve the fuel efficiency of various vehicles and spacecrafts but also reduce carbon dioxide emissions [9]. Therefore, PMCHSs have been developed rapidly [8,10,11,12]. Jiang et al. [13] optimized the performance of PMCHSs with a hollow spring wire by changing the inner diameter of the spring wire. The results showed that when PMCHSs had the same weight as the experimental sample of Liu et al. [14], the spring constant was increased by 11.12%; by contrast, when PMCHSs had the same spring constant as the experimental sample, the weight was reduced by 8.85%. Zhan et al. [15] optimized PMCHSs under the premise that the spring constant meets the design requirements. The mass of the optimized PMCHS was 8.3% lighter than that of the pre-optimized PMCHS and 34.4% lighter than that of a metal spring with the same spring constant. This shows that optimized PMCHSs can further reduce the mass while ensuring the design requirements. Chiu et al. [16] optimized PMCHSs by changing reinforcement. The results showed that the PMCHS with a rubber core could increase its failure load in compression by about 12%. The PMCHS with a braided outer layer could not only increase its failure load in compression by about 18% but also improve the spring constant by approximately 16%. Sui et al. [17] studied the resilience of PMCHSs with multistrand reinforcement and discussed the influence of the inner diameter of PMCHSs, the number of fiber strands, and the twist of reinforcement on their resilience. The results showed that the resilience performance of the PMCHS was gradually optimized with the decrease in the inner diameter and the increase in the number of fiber strands and twist. Hwan et al. [18] optimized the fatigue properties of PMCHSs with rubber core diameters of 3 mm, 4 mm, 5 mm and braided outer layers of 1, 2, 3, respectively. The results showed that the fatigue performance increased with the decrease in the diameter of the rubber core based on the fix braided outer layers. Sancaktar et al. [19] investigated the effects of the number of active coils and the ratio of the diameters of PMCHSs to the diameter of the spring wire (D/d) on the fundamental natural frequencies of PMCHSs with circular cross sections. Their experimental results revealed that the natural frequency of PMCHSs can be lowered by increasing the D/d ratio and the number of turns. It can be seen from the above research that the static and dynamic properties of PMCHSs have been gradually optimized. However, the above research only studied the single static or dynamic performance of PMCHSs; the relationship between the different properties of PMCHSs have not been considered.

In this work, the torsional property of polymer matrix composite spring wire rods (PMCRs) and the compression and resilience properties of PMCHSs were evaluated. Then, the relationship between those performances was explored, for the first time, to simplify the research method for promoting the development of PMCHSs.

## 2. Design and Experiment of PMCHSs

### 2.1. Design of PMCHSs

**Figure 1:**
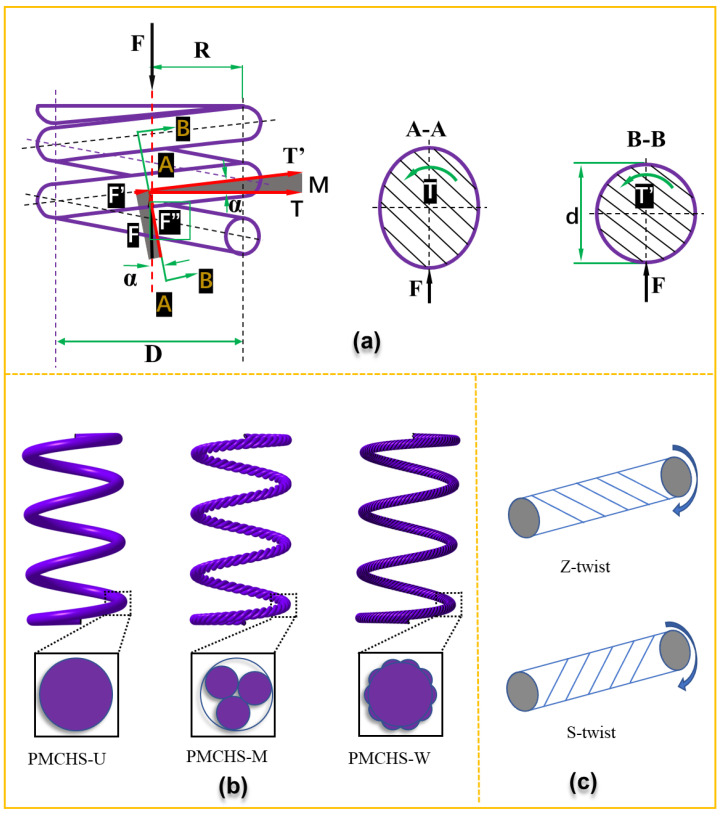
(**a**) Force analysis of a PMCHS under compression with the cross-sectional information in A–A and B–B, (**b**) PMCHSs with unidirectional, multistrand, and wrapped reinforcement structures (PMCHS-U, PMCHS-M, and PMCHS-W), and (**c**) force analysis of reinforcement with different twist directions, i.e., Z-twist (the direction of shear stress is opposite to the twist direction) and S-twist (the direction of shear stress is the same as the twist direction).

When a PMCHS is subjected to an axial load F, the forces/moments acting on the axial section A–A include axial force F and torque moment T, and the forces/moments acting on the normal cross-section B–B include transversal force F”, axial force F’, torque moment T’ and bending moment M, as shown in Figure 1a. When the helix angle α is small and the spring winding ratio (spring index) is large, the bending moment M, axial force F’, and transversal force F” contribute little to the stiffness of the spring [1]. It is sufficient to consider only the torque T of the polymer matrix composite spring wire rods (PMCRs); therefore, the property of PMCHSs is related to that of PMCRs. In addition, the spring constant of a PMCHS can be expressed by Equation (1) [10]:

(1) $$k=\frac{Gd^{4}}{8nD^{3}}$$

where *G* is the shear modulus, *D* is the diameter of the spring, d is the spring wire diameter, and n is the number of coil turns. 

As shown in Equation (1), the spring constant can be changed by changing the shear modulus based on a fixed geometry. The shear modulus can be adjusted effectively by changing the structure of the spring reinforcement. Three kinds of PMCHSs with the reinforcement structures of unidirectional, multistrand, and wrapped (PMCHS-U, PMCHS-M, and PMCHS-W) were proposed by combining them with textile structures, as shown in Figure 1b. The reinforcement of PMCHS-U was that each fiber bundle was arranged in parallel without any entanglement and twisting between the fiber bundles. The reinforcement of PMCHS-M was to twist several bundles of fibers into a composite yarn. The reinforcement of PMCHS-W was to add the fiber bundles as the core yarn on the basis of the reinforcement of PMCHS-M. As the twist direction of the reinforcement should be designed according to the rotation direction of PMCHSs to enhance its contribution, it is necessary to determine the twist direction of the reinforcement before preparation. The shear stress at the right end face of the spring wire with any length acts clockwise in the right helical compression spring. Therefore, when the twist direction of a reinforcement structure is Z-twist, it means the direction of the shear stress is opposite to the twist direction; when the twist direction of a reinforcement structure is S-twist, it means the direction of the shear stress is the same as the twist direction, as shown in Figure 1c. However, reinforcements with multistrand or wrapped structures can maximize their torsional properties only when the twist direction is the same as the direction of the shear stress. Otherwise, the untwisting phenomenon will occur, which means the torsion performance will be significantly reduced. Therefore, the twist direction in the multistrand and wrapped structures is the S-twist direction.

### 2.2. Experimental

#### 2.2.1. Materials

The reinforcement structure of the PMCHSs was made of carbon fiber (T700SC-12 K, Toray, Tokyo, Japan). The resin and hardener used to cure the PMCHSs were GCC-135 epoxy resin and GCC-137 curing agent (Kunshan Lvxun chemical Ltd. Co. Jiangsu, China). All the materials were used without further treatments and were used in our previous research [20].

#### 2.2.2. Manufacturing PMCRs and PMCHSs

**Figure 2:**
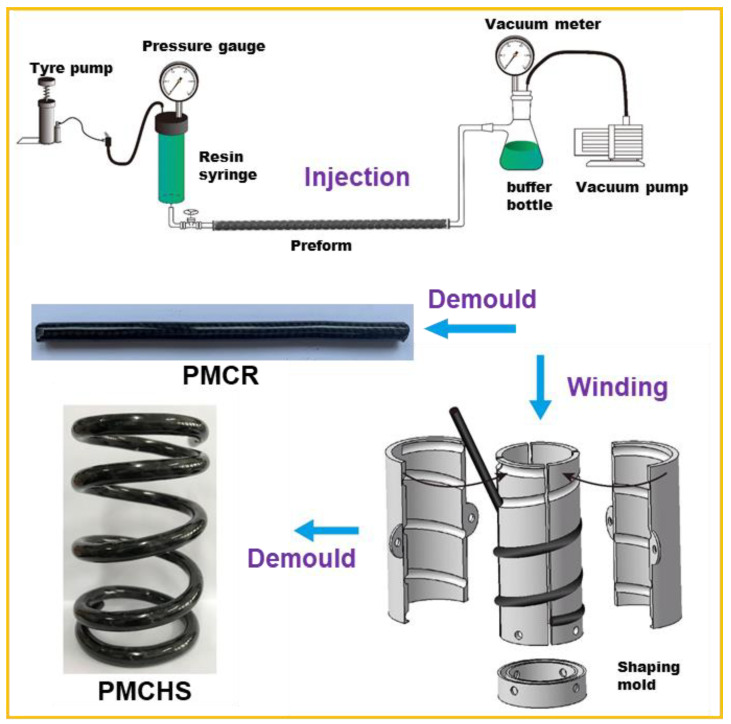
Schematical drawing of manufacturing PMCRs and PMCHSs based on an injection–winding technology.

The PMCRs were manufactured via an injection after inserting a reinforcement structure into a PVC tube. The first step was the preparation of a preform. The preparation process is shown in Figure 2. The mixture made of epoxy resin GCC-135 and curing agent GCC-137 in the volume ratio of 3: 1 was poured into a resin syringe. A pressure pump and a vacuum pump were opened, and the pressure gauge and vacuum meter were set to 0.3 MPa and 0.09 MPa, respectively; then, the mixture entered the preform at a constant speed and slowly permeated the preform. When there were a few mixture bubbles flowing into a buffer bottle, the pressure and vacuum pumps were closed, and the preform was removed after sealing both ends of the preform. One end of the preform was suspended and fixed, and the other end hung a heavy object to further straighten the fibers in the preform and reduce the internal stress caused by the irregular buckling of the fibers. After that, the preform was cured at room temperature for 48 h. Finally, the PMCRs with unidirectional, multistrand, and wrapped textile structural reinforcement (abbreviated as PMCR-U, PMCR-M, and PMCR-W) were manufactured after removing the PVC tube. The preparation of PMCHSs only needed to add a winding process based on the preparation of PMCRs, that is, a PMCR before curing was wound on a shaping mold; after curing, PMCHS-U, PMCHS-M, and PMCHS-W (*α* = 5°, *D* = 63 mm, n = 5, d = 6 mm) with smooth surfaces were obtained by removing the mold and the PVC tube.

#### 2.2.3. Fiber Volume Content (*V*_f_)

The fiber volume content (*V*_f_) of the samples was evaluated according to ASTM D3171. Each sample with approximately 5 g was first put into a 30 mL nitric acid solution (concentration > 90%) at a constant temperature of 75 ± 1 °C (in an oven) for 5 h. The specimen was taken out and then washed with acetone and distilled water, separately. Then, it was put back in the oven at 100 ℃ for 0.5 h. Finally, it was taken out. The *W*_f_ and *V*_f_ can be calculated separately as follows [16]:

(2) $$W_{f}=\frac{W_{1}}{W_{2}}\times 100\%$$

(3) $$V_{f}=\frac{W_{f}/\rho_{f}}{W_{f}/\rho_{f}+W_{r}/\rho_{r}}\times 100\%$$

where *W*_f_ is the fiber weight fraction (%), *W*_1_ is the weight of the fiber (g), *W*_2_ is the weight of the specimen (g), *W*_r_ is the resin weight fraction, (%) *ρ*_f_ is the density of the fiber (g/cm^3^), and *ρ*_r_ is the density of the resin (g/cm^3^).

It can be seen from Table 1 that the difference between the experimental and theoretical values of PMCRs is negative, while that of the PMCHSs is positive. This is because the pressure inside a PVC tube during the preparation of PMCRs increased the volume of the PVC tube, resulting in a smaller actual *V*_f_. However, when the PMCHSs were wound on the mold, the resin flowed to both ends of the PVC tube. The excess resin at both ends of the PVC tube was cut off during post-treatment, resulting in a larger actual *V*_f_. The maximum difference among all the samples was 0.93%, indicating that the preparation process was stable and controllable.

### 2.3. Mechanical Property Characterizations 

#### 2.3.1. Torsion Test of PMCRs

**Figure 3:**
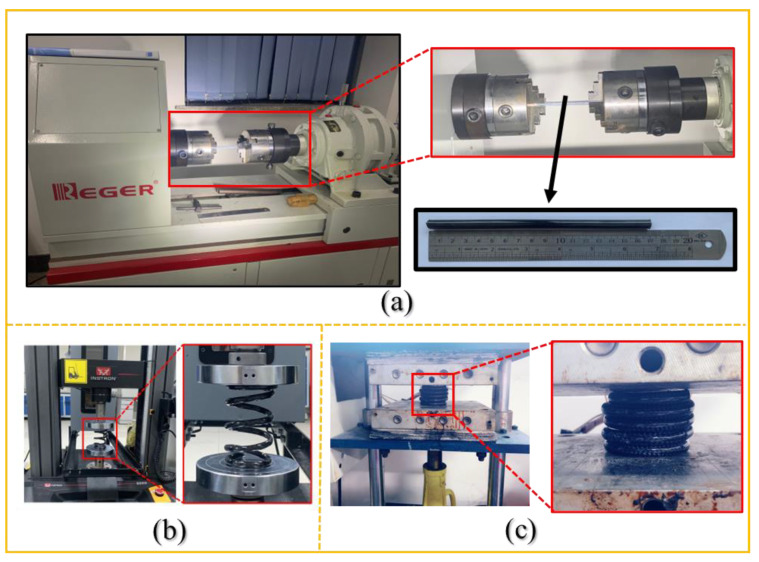
Test setups for (**a**) torsion test of PMCRs, (**b**) compression of PMCHSs, and (**c**) resilience of PMCHSs.

An Rnj-500 microcomputer-controlled torsion testing machine was used to characterize the torsion property of the PMCRs according to ASTM D5279, as shown in Figure 3a. The sample length was 17 cm, the test gauge distance was 8 cm, and the test speed was 40 °/min. Torsion properties in the same and opposite directions with the twist direction of each PMCR were tested. Five samples were tested for each PMCR.

#### 2.3.2. Compress Properties of PMCHSs

The compression test of the PMCHSs was conducted by using an Instron 5969 strength machine according to ASTM A 125-2001, as shown in Figure 3b. The test speed was 10 mm/min, and the compression height was 70% of the free height of a PMCHS. The test system automatically recorded the data of the applied force and the corresponding compression amount in each sampling interval during the test, to immediately provide the load–displacement curve of the samples. Five samples were tested for each PMCHS.

#### 2.3.3. Resilience of PMCHSs

The resilience test was conducted by pressing a PMCHS until a mutual contact between the coil levels was achieved [12], as shown in Figure 3c, and then keeping the compression load for a period of 24 h, 48 h, 72 h, and 96 h, respectively. The spring’s height after each loading period was recorded; then, the spring height after unloading for 24 h, 48 h, 72 h, and 96 h was measured and recorded. Five samples were tested for each PMCHS.

## 3. Results and Discussion

### 3.1. The Torsion Property of PMCRs

**Figure 4:**
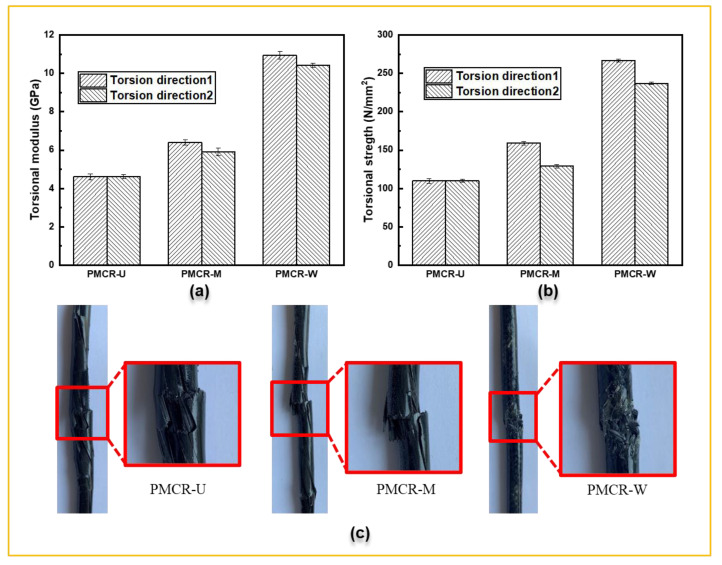
(**a**) Torsional modulus and (**b**) torsional strength of PMCRs with different torsional directions, i.e., torsional direction 1 (the torsion direction was the same as that of the twist direction of reinforcement) and torsional direction 2 (the torsion direction was opposite to that of the twist direction of reinforcement), and (**c**) failure topographies of PMCRs.

The PMCHSs were made by winding the PMCRs on a shaping mold with helical grooves, indicating mechanical properties of a PMCHS are related to the mechanical properties of the corresponding PMCR. Therefore, the mechanical properties of the PMCRs were analyzed before studying the properties of the PMCHSs. It can be seen from Figure 4a that the torsion modulus of PMCR-W was the largest. The difference between the wrapped structure and the other two types was obvious, while the difference between PMCR-U and PMCR-M was small. When the torsion direction was the same as that of the twist direction of the reinforcement, the torsion moduli of PMCR-W and PMCR-M were 137.1% and 38.7% larger than that of PMCR-U, respectively. When the torsion direction was opposite to that of the twist direction of the reinforcement, the torsion moduli of PMCR-W and PMCR-M were 126.0% and 27.9% larger than that of PMCR-U, respectively. It is obvious that when the torsion direction was opposite to the twist direction of the reinforcement structure, the torsion modulus was slightly reduced. The reductions in PMCR-M and PMCR-W were 7.8% and 4.7%, respectively. In addition, Figure 2 shows that the torsional strength of PMCR-U, PMCR-M, and PMCR-W decreased in turn. The torsional strength values of PMCR-W and PMCR-M were 142.8% and 44.6% higher than that of PMCR-U, respectively, when the torsion direction was the same as the twist direction. When the torsion direction was opposite to the twist direction, the torsional strength was significantly reduced. The reductions in PMCR-M and PMCR-W were 18.9% and 11.2%, respectively. The twist direction has a great impact on the performance of PMCRs. Therefore, the twist direction and the spring rotation direction must be matched during manufacturing PMCHSs. Figure 4c shows the failure morphologies of PMCR-U, PMCR-M, and PMCR-W when the torsion direction was the same as the twist direction. All the reinforcement fibers in PMCR-U were arranged parallelly. Therefore, the friction coefficient between the external surface of the reinforcement and the resin was small, and the interface performance was poor. The fibers and resin were de-bonded, and the fibers were gradually loose and broken during the torsion test, indicating weak mechanical properties. By contrast, the twist structure of PMCR-M made the fibers close to each other to increase its mechanical properties [20]. When the twist angle reached a certain value, the fibers showed a state of transverse shear cracking. The surface roughness of the reinforcement of PMCR-W led to its better interface performance, and the outer layer wrapped structure of PMCR-W could obviously increase its torsional performance [21]. Therefore, only the resin was destroyed when PMCR-W failed.

### 3.2. Compression Evaluations of PMCHSs

**Figure 5:**
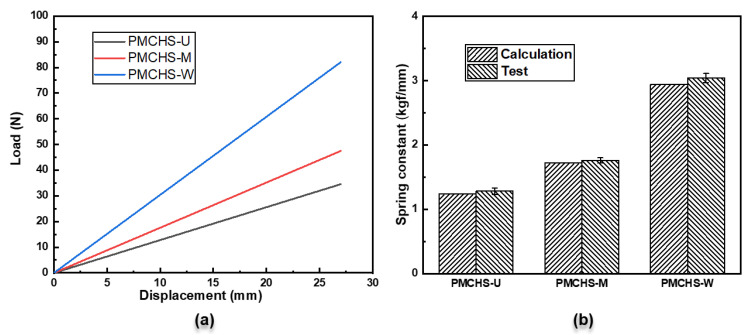
(**a**) Load–displacement curves of PMCHSs and (**b**) comparison of calculated and experimental spring constants.

The load–displacement curves of the PMCHSs after compression are shown in Figure 5a. The results showed that the loads of PMCHS-U, PMCHS-M, and PMCHS-W at the maximum displacement were 34.6 N, 47.5 N, and 82.1 N, respectively. The load of PMCHS-M was 37.5% higher than that of PMCHS-U. The ability to withstand the multiaxial loads of PMCHS-M can be enhanced after the fiber direction is changed by twisting [16]. The twist direction of the reinforcement was the same as the torsion direction in PMCHS-M. The reinforcement fibers of PMCHS-M were tightly held together under the effect of the torsion moment during compression. Therefore, the force was easily transmitted and uniformly dispersed among the fibers. The optimization effect of PMCHS-W was obvious, the load of PMCHS-W was 72.6% and 137.5% higher than that of PMCHS-M and PMCHS-U, respectively. This is because the torsional shear stress increased with the increase in the spring wire diameter [1], which means that increasing the performance level of the outer surface of the spring wire is the most effective way for improving spring performance. Therefore, the performance of PMCHS-W with an optimized spring wire peripheral structure can greatly improve. In addition, since PMCHSs are mainly subjected to torsion during compression, it can be stated that the torsion modulus of a PMCR is approximately equal to the shear modulus of the corresponding PMCHS. Therefore, the relationship between the performance of a PMCR and the corresponding PMCHS can be established. The spring constant of a PMCHS obtained by compression was compared with that calculated by the torsion modulus of the corresponding PMCR based on the spring stiffness formula, as shown in Figure 5b. The spring constant test results of PMCHS-U, PMCHS-M. and PMCHS-W were slightly larger than the calculation results, because the calculation results ignored the bending moment, axial force, and shear force of the springs [22]. The differences between the two spring constants of PMCHS-U, PMCHS-M, and PMCHS-W were 3.1%, 2.3%, and 3.3%, respectively, which indicates that the spring constant values of the PMCHSs could be obtained by using the torsion moduli of the corresponding PMCRs. The proven relationship between the torsion property of PMCRs and the compression property of the corresponding PMCHSs can significantly simplify the property study of PMCHSs.

### 3.3. Resilience of PMCHSs

**Figure 6:**
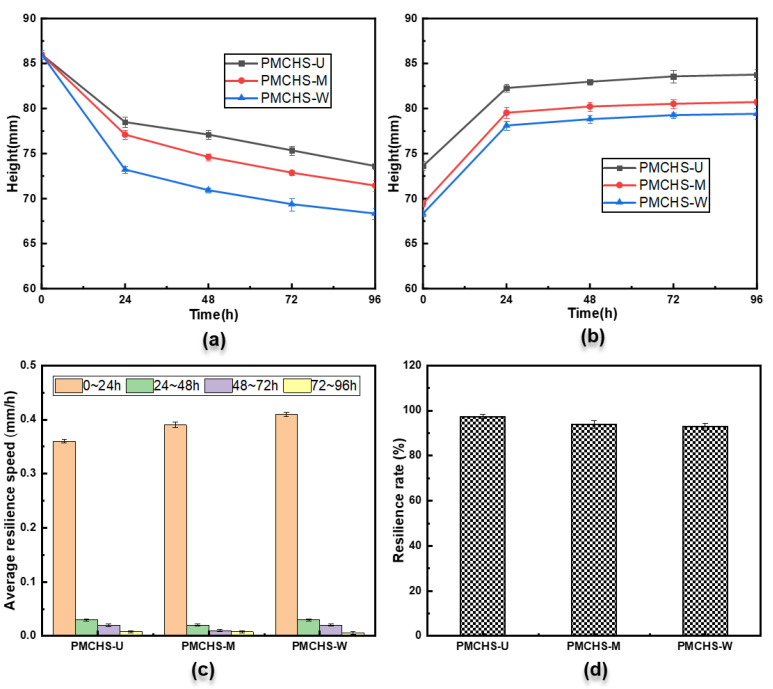
(**a**) Time–height curves of three types of PMCHSs during loading, (**b**) time–height curves of three types of PMCHSs during unloading, (**c**) resilience speed of three types of PMCHSs, and (**d**) resilience rate of three types of PMCHSs.

PMCHSs are constantly compressed during operation, so the resilience performance also needs to be further studied. The comparison of the resilience performance of the three PMCHSs is shown in Figure 6. It can be seen from Figure 6a that the changing trend of the heights of the three PMCHSs was the same, that is, the spring height reduced with the increase in the loading time. The heights of PMCHS-U, PMCHS-M, and PMCHS-W decreased by 8.7%, 10.4%, and 14.9% after 24 h, respectively, implying that the deformation of PMCHS-U, PMCHS-M, and PMCHS-W consequently increased. This is because the scattered internal reinforcement structural fibers in PMCHS-U made the force capacity small; hence, the damage degree of the resin was small due to less force acting on the resin. The entanglement between the reinforcement fibers of PMCHS-M and PMCHS-W improved their mechanical properties; hence, the damage degree of the resin was high due to the large force acting on the resin. The decreasing trend of the spring height showed a gradual reduction; the heights of PMCHS-U, PMCHS-M, and PMCHS-W were 73.6 mm, 71.4 mm, and 68.3 mm after 96 h, respectively. Then, the heights of the three springs continuously increased with the same trend after unloading, as shown in Figure 6b. It can be seen from Figure 6c that the average resilience speed (the ratio of the height difference in PMCHSs to the corresponding test time difference) values of PMCHS-U, PMCHS-M, and PMCHS-W in the first 24 h increased in order, which were 0.36 mm/h, 0.39 mm/h, and 0.41 mm/h, respectively. This is mainly because PMCHS-M and PMCHS-W have large energy storage due to the role of their internal reinforcement, which made them more likely to return to their original state immediately after unloading. The resilience speed of each spring gradually decreased by 0 with the increase in time. Figure 6d shows the final resilience rate of the three springs, that is, the ratio of the final height after unloading for 96 h to the initial height. The resilience rates of PMCHS-U, PMCHS-M, and PMCHS-W were consequently reduced, with 97.4%, 93.9%, and 92.8%, respectively. Compared with the results of the above compression performance, it can be concluded that the resilience performance of the PMCHSs decreased with the increase in the spring constant. This is because the toughness of the resin in PMCHSs was poor, the damage degree of the resin increased with the increase in the spring constant during the compression process, and the resilience decreased with the increase in the damage degree of the resin.

### 3.4. Prediction of Comparison between the PMCHSs and Steel Spring

**Figure 7:**
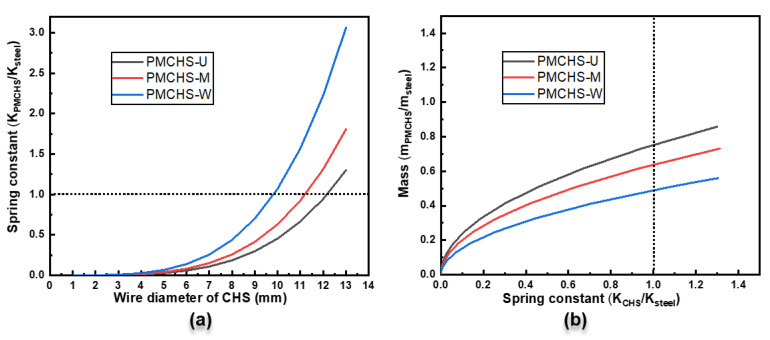
(**a**) The spring constant ratios of different PMCHSs to a steel spring with the increase in the wire diameter of the PMCHSs, and (**b**) the mass ratio of different PMCHSs to a steel spring with the increase in the spring constant ratio. The dot line means the PMCHS and steel springs having the same spring constant.

To further understand the difference between PMCHSs and steel springs, their performances were compared. The performance of PMCHS-U, PMCHS-M, and PMCHS-W could be adjusted by changing the diameter of the spring, the diameter of the spring wire, and the number of coil turns without changing the structural parameters of the reinforcement. However, the change in the diameter of the spring and the number of coil turns affects the stress state of PMCHSs [1], so in this study, we only compared the performance of the PMCHSs with that of the steel spring by changing the wire diameter of the PMCHSs. The size of the steel spring was the same as that of the PMCHSs manufactured in this experiment, and the shear modulus of the steel spring was 78.5 GPa. The spring constant ratio of a PMCHS to a steel spring is determined by Equation (1) and the mass ratio of a PMCHS to a steel spring is determined by Equations (2) and (3), which are shown in Figure 7. The dotted line in Figure 7 indicates that the spring constant of the PMCHSs was equal to that of the steel spring. It can be seen from Figure 7a that the spring constant ratio of the PMCHSs to the steel spring rapidly increased with the increase in the wire diameter of the PMCHSs. When the wire diameters of PMCHS-U, PMCHS-M, and PMCHS-W were 12.16 mm, 11.18 mm, and 9.81 mm, respectively, their spring constant values were the same as that of the steel spring. When the wire diameters of PMCHS-U, PMCHS-M, and PMCHS-W were 13 mm, the values of the spring constant reached 1.30, 1.81, and 3.06 times that of the steel spring, respectively. In addition, the PMCHSs had a greater mass advantage than the steel spring. It can be seen from Figure 7b that the mass ratio of the PMCHSs to the steel spring slowly increased with the increase in the spring constant ratio. When the spring constant of PMCHS-U, PMCHS-M, and PMCHS-W was equal to that of the steel spring, their masses only reached 75%, 63%, and 49% of the mass of the steel spring, respectively.

## 4. Conclusions

In this study, PMCHS-U, PMCHS-M, and PMCHS-W were designed, manufactured, and evaluated. The torsion of the PMCRs and the compression and resilience of the PMCHSs showed that PMCHS-M and PMCHS-W could effectively improve the performance of the PMCHSs. The compression capacity of PMCHS-W was 72.6% and 137.5% higher than that of PMCHS-M and PMCHS-U, respectively. The spring constant values obtained by using the torsion modulus of the PMCRs were only 3.1%, 2.3%, and 3.3% different from the compression test results from the corresponding PMCHSs, respectively. The resilience results showed that the resilience performance of the PMCHSs decreased with the increase in the spring constant. Finally, the advantages of the PMCHSs were highlighted by comparing them with a steel spring. When the spring constant values of PMCHS-U, PMCHS-M, and PMCHS-W were equal to that of the steel spring, their masses were only 75%, 63%, and 49% of the mass of the steel spring, respectively. The proposed PMCHSs can provide a lightweight reference for the springs used in engineering applications.

## Figures and Tables

**Table 1:** The comparison of theoretical values and actual *V*_f_ values of PMCRs and PMCHSs.

Types	Theoretical Value	Actual Value
PMCR-U	50.00%	49.40%
PMCR-M	50.00%	49.47%
PMCR-W	50.00%	49.53%
PMCHS-U	50.00%	50.62%
PMCHS-M	50.00%	50.93%
PMCHS-W	50.00%	50.74%

## Data Availability

Not applicable.

## References

[1] Ke J., Wu Z.Y., Liu Y.S., Xiang Z., Hu X.D. (2020). Design method, performance investigation and manufacturing process of composite helical springs: A review. Compos. Struct..

[2] Ekanthappa J., Basavarajappa S., Sogalad I. Investigation on design and fabrication of continuous fiber reinforced composite helical spring for automobile suspension. Proceedings of the National Conference on Challenges in Research & Technology in the Coming Decades.

[3] Shinde R.M., Godase S.P., Sawant S.M. Comparison of weights of steel and composite coil spring for two wheeler suspension systems. Proceedings of the 4th RIT Post Graduates Conference.

[4] Londhe A.B. (2013). FEA and analytical analysis of natural fibers composite leaf spring. Int. J. Mech. Eng. Res..

[5] Kara Y. (2017). A review: Fiber reinforced polymer composite helical springs. Mater. Sci. Nanotechnol..

[6] Shokrieh M.M., Rezaei D. (2003). Analysis and optimization of a composite leaf spring. Compos. Struct..

[7] Stephen C., Selvam R., Suranjan S. (2019). A comparative study of steel and composite helical springs using finite element analysis. Advances in Science and Engineering Technology International Conferences (ASET).

[8] Chen L., Xing W., Wu L., Chong J., Lei T., Jiang Q., Tang Y. (2022). Understanding multiple parameters affecting static and dynamic performances of composite helical springs. J. Mater. Res. Tech..

[9] Ghassemieh E. (2011). Materials in automotive application, state of the art and prospects. New Trends Dev. Auto. Ind..

[10] Ashok N.A., Kumar R., Singh M., Singh J., Gulati P., Singh J. (2021). Development method, manufacturing process of fibre reinforced polymer composite type helical springs: A review. Rec. Trends Eng. Des..

[11] Jang D., Jang S. (2014). Development of a Lightweight CFRP Coil Spring.

[12] Kumar M.S., Vijayarangan S. (2007). Static analysis and fatigue life prediction of steel and composite leaf spring for light passenger vehicles. J. Sci. Ind. Res. India.

[13] Bai J.B., Liu T.W., Wang Z.Z., Lin Q.H., Cong Q., Wang Y.F., Ran J.N., Li D., Bu G.Y. (2021). Determining the best practice–optimal designs of composite helical structures using genetic algorithms. Compos. Struct..

[14] Liu T.W., Bai J.B., Lin Q.H., Cong Q. (2021). An analytical model for predicting compressive behaviour of composite helical structures: Considering geometric nonlinearity effect. Compos. Struct..

[15] Zhan B., Sun L., Huang B., Zhao G., Wang Q. (2018). Design and optimization of automotive composite helical spring. J. Beijing Univ. Aer. Ast..

[16] Chiu C.H., Hwan C.L., Tsai H.S., Lee W.P. (2007). An experimental investigation into the mechanical behaviors of helical composite springs. Compos. Struct..

[17] Sui G., Fan Y., Zhong W., Zhang Z., Sun Z., Chen R. (2001). Manufacture and experiment study of composite cylindroid spiral spring. Acta Mater. Compos. Sin..

[18] Hwan C.L., Chiu C.H., Lee W.L., Lee W.P. (2010). The effects of rubber core diameter and the number of braided outer layers on the compression and fatigue properties of helical composite springs. J. Adv. Mater..

[19] Sancaktar E., Gowrishankar S. Natural frequencies of composite cylindrical helical springs manufactured using filament winding. Proceedings of the International Design Engineering Technical Conferences and Computers and Information in Engineering Conference.

[20] Wu L., Chen L., Fu H., Jiang Q., Wu X., Tang Y. (2020). Carbon fiber composite multistrand helical springs with adjustable spring constant: Design and mechanism studies. J. Mater. Res. Tech..

[21] Jiang Q., Qiao Y., Zhao F., Pan Z., Wu X., Wu L., Fu H. (2021). Composite helical spring with skin-core structure: Structural design and compression property evaluation. Polym. Compos..

[22] Xiong Z., Song R., Kang Z., Liu L., Liu Q. (2015). Analysis on rigidity of composite helical spring and its influence factors. Eng. Mech..

